# The intrinsic and synaptic responsiveness of a new realistic Purkinje cell model

**DOI:** 10.1186/1471-2202-14-S1-P80

**Published:** 2013-07-08

**Authors:** Stefano Masoli, Sergio Solinas, Egidio D'Angelo

**Affiliations:** 1 Department of Brain and Behavioural Science, Neurophysiology and Neurocomputation Unit, University of Pavia, Via Forlanini 6, I-27100, Pavia, Italy; 2 Brain Connectivity Center, Istituto Neurologico IRCCS C. Mondino, Pavia, I-27100, Italy

## 

The latest discoveries on Purkinje cell (PC) physiology suggest that the mechanisms of PCs intrinsic excitability have to be revisited. Starting from available models [1], we have constructed a new PC model in Python-NEURON, which explicitly accounts for the Axon Initial Segment (AIS) [2-4] and a part of the axon including the first node of Ranvier (RVN). The fast Na+ channels are located in AIS, soma with initial dendrite and RVN [4]. The K+ delayed rectifier channels are located only in the soma. The Ca2+ and Ca2+-dependent K+ channels, including SK2, as well as intracellular Ca2+ dynamics have been updated [5]. The new model configuration now generates simple spike (SS) firing reproducing the experimental input-output curve[6]. SSs initiate in AIS and then back-propagate into the soma decaying sharply inside the dendritic tree. Activation of parallel fiber (pf) generates a short burst followed by a pause caused by Stellate cells. Following a complex spike (CS), SS activity is interrupted independently of the inhibitory synaptic input. Interestingly, the model can shift its state from silent to autorhythmic (configuring a bistable behavior) upon transient current injection or activation of CFs. The pf and granule cell ascending axon (aa) synapses have been modeled using a stochastic release mechanism activating AMPA synaptic receptors. The facilitation and depression profiles of pf and aa synapses faithfully reproduce the experimental data. This model provides a valuable tool to further investigate the Purkinje cell function in cerebellar network models.
